# Flexibility and small pockets at protein–protein interfaces: New insights into druggability

**DOI:** 10.1016/j.pbiomolbio.2015.01.009

**Published:** 2015-10

**Authors:** Harry Jubb, Tom L. Blundell, David B. Ascher

**Affiliations:** Department of Biochemistry, Sanger Building, University of Cambridge, Tennis Court Road, Cambridge CB2 1GA, UK

**Keywords:** Protein–protein interfaces, Hotspots, Inhibitors druggability

## Abstract

The transient assembly of multiprotein complexes mediates many aspects of cell regulation and signalling in living organisms. Modulation of the formation of these complexes through targeting protein–protein interfaces can offer greater selectivity than the inhibition of protein kinases, proteases or other post-translational regulatory enzymes using substrate, co-factor or transition state mimetics. However, capitalising on protein–protein interaction interfaces as drug targets has been hindered by the nature of interfaces that tend to offer binding sites lacking the well-defined large cavities of classical drug targets. In this review we posit that interfaces formed by concerted folding and binding (disorder-to-order transitions on binding) of one partner and other examples of interfaces where a protein partner is bound through a continuous epitope from a surface-exposed helix, flexible loop or chain extension may be more tractable for the development of “orthosteric”, competitive chemical modulators; these interfaces tend to offer small-volume but deep pockets and/or larger grooves that may be bound tightly by small chemical entities. We discuss examples of such protein–protein interaction interfaces for which successful chemical modulators are being developed.

## 1 Introduction

Multiprotein assemblies mediate the majority of cellular processes, including receptor activation, signal transduction, DNA replication, recombination and repair, and other regulatory events that require high signal-to-noise in cell regulation. Multiprotein assemblies often arise from initial weak binary interactions followed by cooperative, higher-order complex formation, giving high selectivity while at the same time being transient as required for termination of regulatory signals (Higueruelo et al., 2013a).

Multiprotein regulatory systems are assembled mainly through protein–protein interactions (PPIs). Whereas enzyme superfamilies that mediate many signalling events may number hundreds of homologues in the human genome – more than 500 protein kinases and over 600 putative E3 ubiquitin (Ub) ligases (Li et al., 2008) – multiprotein regulatory systems differ widely across each superfamily. The specificity of PPIs offers potential for the development of chemical and biological modulators that target specific pathways, with advantages of selectivity that tend to be difficult to achieve through inhibitors of members of enzyme superfamilies, which tend to be mechanism based, targeting transition/intermediate states or co-factor-binding sites that are similar across the superfamily (Bolanos-Garcia et al., 2012).

Using criteria derived from retrospective analyses of successful drugs, protein–protein interaction sites have historically been described as undruggable (Hopkins and Groom, 2002). Indeed, many protein–protein interfaces, especially those in obligate complexes such as homo-oligomers for the presence of which usually improves stability, have been viewed as large, flat and featureless, and thus difficult targets for the development of small molecule antagonists (Blundell et al., 2000, Blundell et al., 2006, Jones and Thornton, 1996). With the wealth of information available from structural biology programmes, and advances in experimental and computational assessment of druggability, this traditional view of protein–protein interaction interfaces is being reassessed (Kastritis and Bonvin, 2013, Loving et al., 2014, Villoutreix et al., 2014), presenting new insights for the development of “orthosteric” PPI modulators that compete for the binding-site surface of a PPI interface, typically with the objective of sterically inhibiting the association of a multiprotein complex.

In this review we highlight the importance of relatively small pockets that can lead to very selective binding at PPI interfaces (Blundell et al., 2006, Jubb et al., 2012, Koes and Camacho, 2012a, Koes and Camacho, 2012b). We show that small, single-residue sub-pockets and regions of surface depth bound by continuously interacting peptide segments extend the concept of druggability in ways peculiar to protein–protein interactions (Ben-Shimon and Eisenstein, 2010, Fuller et al., 2009, Guo et al., 2014, Koes et al., 2012, Kozakov et al., 2011, Li et al., 2004b, London et al., 2010, London et al., 2013, Rajamani et al., 2004, Winter et al., 2012) and provide tractable sites for the development of chemical modulators (Arkin et al., 2014). We posit that interactions involving short peptides, linear binding motifs within larger intrinsically disordered regions or within loops or loop-termini of globular proteins, and possibly linear epitopes arising from surface exposed helices, can provide promising binding sites. The loss of entropy on binding a flexible peptide is likely countered by binding larger sidechains, such as those of tryptophan, tyrosine, phenylalanine or arginine, in distinct preformed pockets (Blundell et al., 2006), or even smaller hydrophobic residues such as alanine in pockets where they may relieve energetically “unhappy” surface waters (Huggins et al., 2011).

## 2 Flexibility in partner interactions

**Fig. 1:**
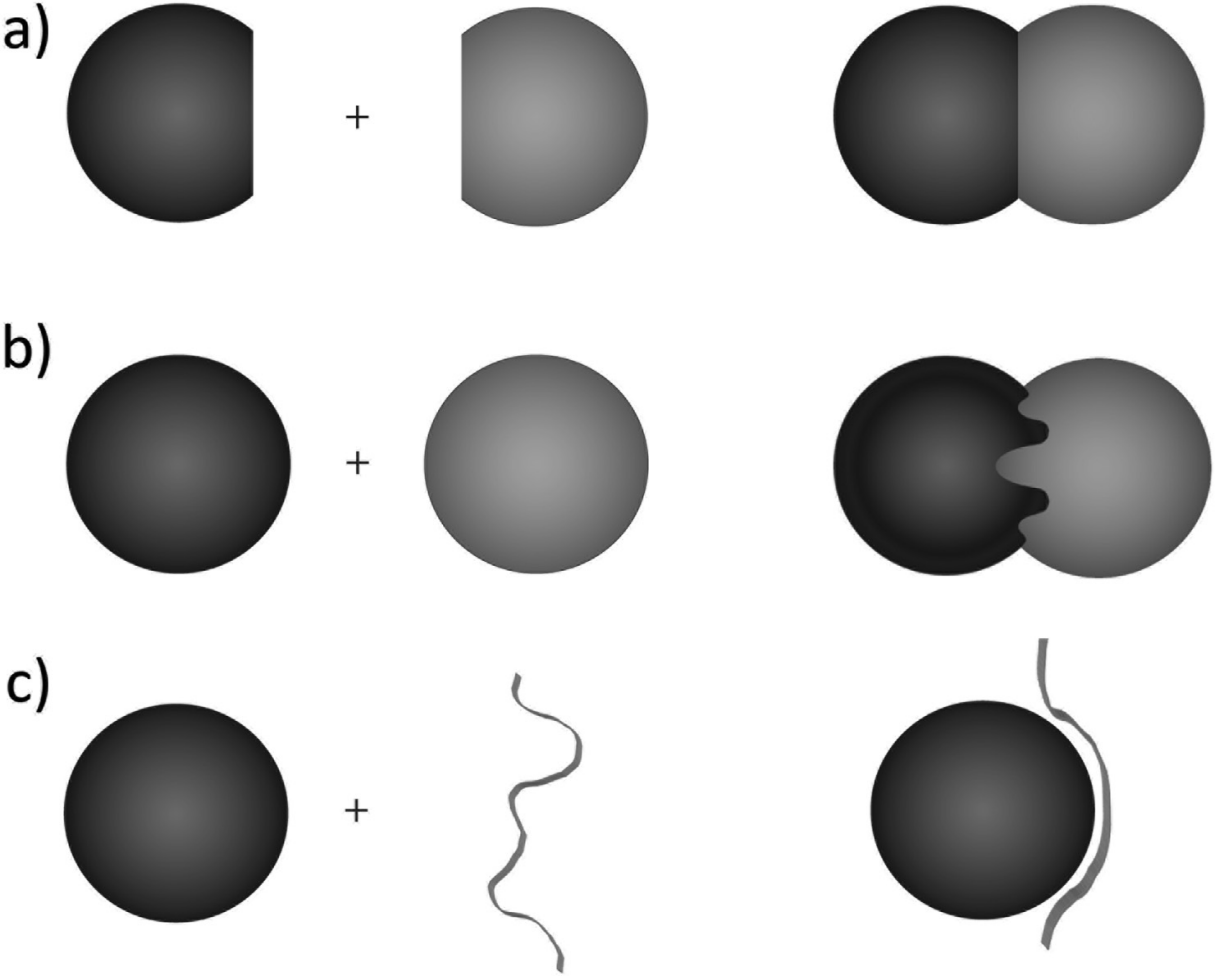
**Three models of binary protein–protein interactions**. (a) Preformed globular structures that interact through a discontinuous epitope with no conformational change. (b) Preformed globular structures that adopt a novel conformation in the complex. (c) Unstructured proteins that fold on binding their partners. Reproduced with permission from Blundell T.L. et al. (2006).

Binary PPIs, which have been targeted in drug discovery and in which different degrees of conformational change and loss of entropy occur on binding, can be described by three models: those where both partners have preformed, relatively rigid structures; those where one or both of the preformed structures undergo significant conformational changes on interaction; and those where one of the structures folds as it binds (Fig. 1) (Blundell and Wood, 1982, Blundell et al., 2006, Pawson and Nash, 2003). There are also some cases where both partners may fold on interaction, but these are relatively uncommon and may less likely provide targets, at least for binding to one of the partners in isolation; for example where homodimers that are expressed simultaneously fold together permanently in an intertwined or interdigitated structure (Bonvin et al., 1994, Kishan et al., 1997). Numerous databases including the 3D Interaction Domains (3DID (Stein et al., 2011); http://3did.irbbarcelona.org/), Domain Annotated Protein–protein Interaction Database (DAPID (Chen et al., 2006); http://gemdock.life.nctu.edu.tw/dapid) and PICCOLO (Bickerton et al., 2011) (http://www-cryst.bioc.cam.ac.uk/piccolo), have documented structural aspects of PPIs and shown that each of these models is quite common; for reviews of structures, lists of databases and tools for studying protein–protein interactions see (Tuncbag et al., 2009, Villoutreix et al., 2013, Winter et al., 2012).

The first two models involve interactions between globular proteins (see Fig. 1). These represent the “traditional” PPI interface, often described as large (∼1500–3000 Å^2^), flat and relatively featureless interfacial surfaces (Blundell et al., 2000, Jones and Thornton, 1996). The view that these interfaces are featureless has been challenged by the discovery that a few amino acids – so-called hotspots (Clackson and Wells, 1995) – may contribute the majority of interaction free energy in many PPI systems, giving reason for some optimism with respect to targeting specific “hot regions” with chemical modulators (Bogan and Thorn, 1998, Clackson and Wells, 1995, Cukuroglu et al., 2014, Wells and McClendon, 2007). It has been proposed that continuously interacting interface “segments” (Jones and Thornton, 1996, London et al., 2013, Pal et al., 2007) may also play a major role in the architecture of globular protein interfaces, for example the interfaces in TEM1-BLIP and EphB4-EphrinB2 (London et al., 2010).

The third model of protein interaction involves a natively unstructured protein that folds upon interaction with another partner. This was proposed for peptide hormones in the 1970s by Robert Schwyzer (Schwyzer et al., 1979) and experimentally exemplified by X-ray analysis and NMR studies of glucagon in the Blundell and Wüttrich labs (Braun et al., 1983, Sasaki et al., 1975) suggesting a disorder-to-order transition on receptor binding from glucagon with a single turn of helix in solution by NMR (Braun et al., 1983) to one with a much longer region defined by X-ray analysis in the trimer (Sasaki et al., 1975) and at lipid interfaces (Braun et al., 1983) and proposed at the receptor (Blundell, 1979, Blundell and Wood, 1982). Subsequently, Wright & Dyson (Wright and Dyson, 1999, Wright and Dyson, 2009) showed that such concerted folding and binding involving peptides or disordered regions of polypeptide chains is actually widespread in intracellular regulatory systems. To obtain a high-affinity interaction, it would be expected that the smaller surface area provided by peptides and small continuous epitopes requires surface pockets to anchor the peptide in order to maximise intermolecular interactions and to benefit entropically from surface water release into bulk solvent.

**Fig. 2:**
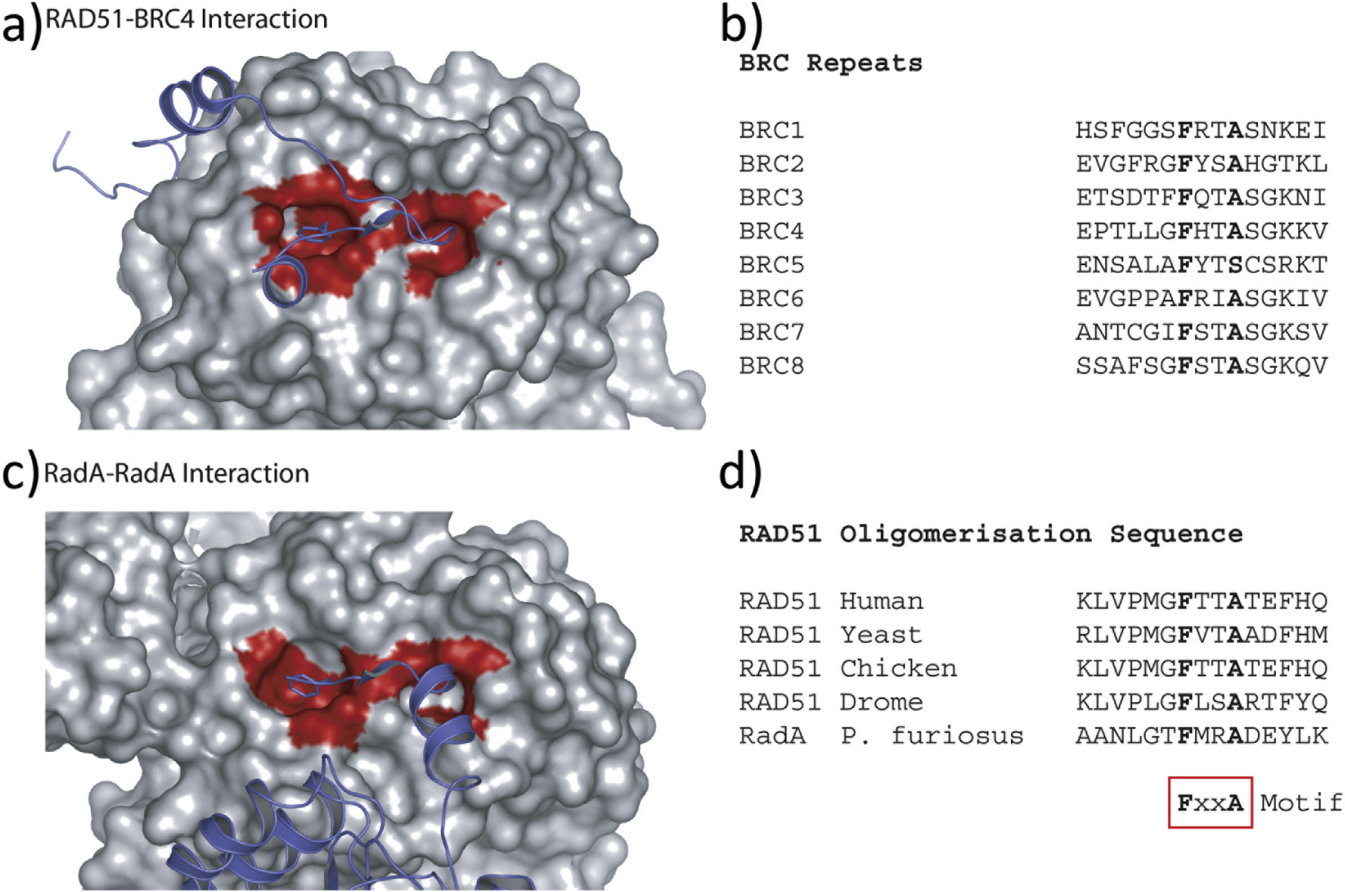
**PPI involving RAD51 and its homologues**. (a) The structure of RAD51 complexed with the region BRC4 of BRCA2 demonstrating the existence of two well-defined pockets on RAD51 that are occupied by side chains of the conserved FxxA motif. RAD51 is shown as grey van-der-Waals surface. BRC4 is shown in purple cartoon form. Residues within a 4 Å radius of the FxxA motif are highlighted in red on the RAD51 surface. (b) Sequences of homologous repeats in human BRCA2. (c) An equivalent view of *P. furiosus* RadA in a protein oligomeric filament showing the similarity of the interface with that of the RAD51 BC4 complex. The interacting oligomerisation region of the adjacent RadA protomer is shown as a purple cartoon. (d) Oligomerisation sequences of RAD51 orthologues and RadA. Reproduced with permission from Winter A. et al. (2012).

An example of a protein–protein interface involving concerted folding and binding of a flexible peptide is the binding of human recombinase Rad51 to BRCA2 in an interaction that is essential for DNA double-strand-break repair through homologous recombination (Pellegrini et al., 2002). The BRC4 peptide found in BRCA2 folds into a defined 3-dimensional structure only upon interacting with Rad51, a disorder-to-order transition (Fig. 2) (Pellegrini et al., 2002). BRCA2 binding disrupts self-association of RAD51 by mimicking RAD51's conserved self-association motif, FxxA (Pellegrini et al., 2002). The conserved phenylalanine of the FxxA motif of BRC4 binds in a deep “anchor” pocket of Rad51, while the conserved alanine binds in a small hydrophobic pocket. Binding to both pockets probably contributes to favourable entropic changes in the system through the release of energetically “unhappy” waters (Huggins et al., 2011).

## 3 The landscapes of pairwise protein–protein interfaces

Drug-like molecules typically exert their actions through binding to high-affinity sites of the right shape and chemical composition. These were traditionally viewed to not be present in the relatively flat and featureless PPI interfaces. Analyses of PPI interfaces using new computational tools can identify key residues in interfaces mediating the protein–protein interaction (Pires et al., 2014) and potential binding sites (Hendlich et al., 1997, Kalidas and Chandra, 2008, Laurie and Jackson, 2005, Morita et al., 2008). Recent studies have shown that successful orthosteric PPI inhibitors do indeed exploit multiple, small volume pockets (Fuller et al., 2009), which often play roles as “anchors” and/or hotspots in the interface (Ben-Shimon and Eisenstein, 2010, Jubb et al., 2012, Li et al., 2004a, Rajamani et al., 2004) and/or are potential fragment binding sites (Jubb et al., 2012, Scott et al., 2013, Zerbe et al., 2012).

**Fig. 3:**
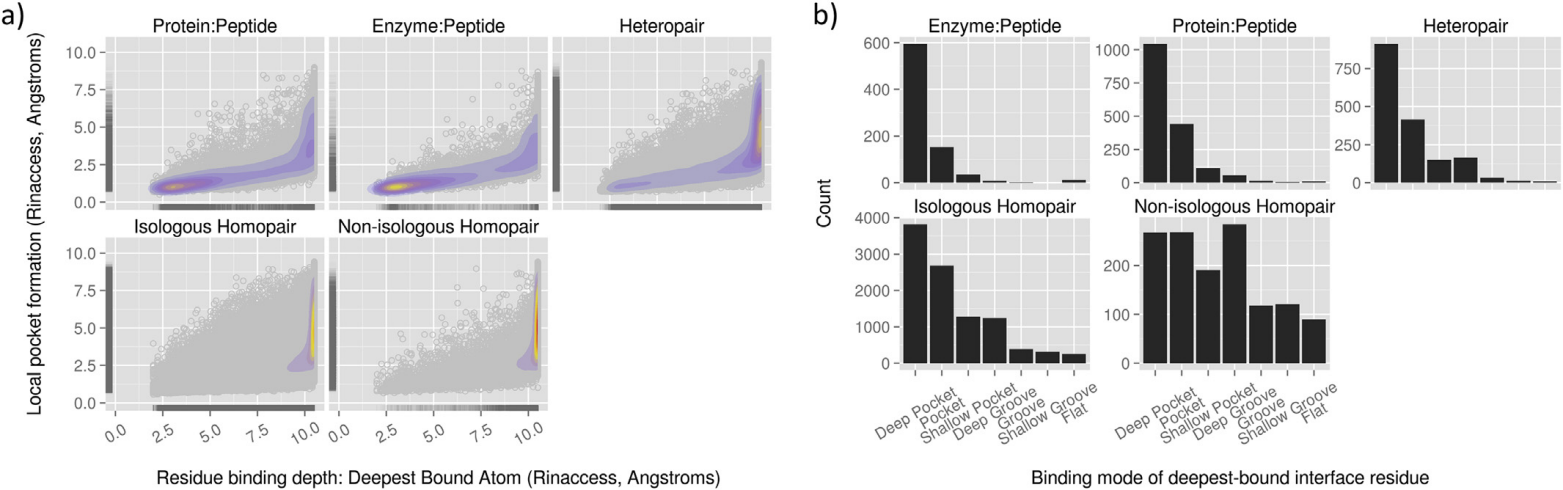
**Residue binding modes at pairwise PPI interfaces**. (a) Comparison of binding site depth utilisation by residues in different classes of pairwise PPI interface. Each point represents a residue contributed by the shortest chains in each interface pair. The abscissa indicates how deeply a residue is bound into the partner protein's surface, measured using R_inaccess_ (see text). The scale ranges from <2.5 Å, which represent deep binding pockets, to 10.5 Å, which represents flatness. The ordinate measures how deep the local pocket environment around the residue is, measured as the deepest partner protein atom found within 5 Å of the residue of interest. The 2D density mapping shows that peptide interfaces proportionally make better use of the concavity available to them, whereas for globular interfaces the majority of interface residues lie flat against binding surfaces of variable depth. (b) Comparison of the binding mode of the deepest bound residues from interfaces of different classes. The ordinate counts the number of interfaces with the deepest interface residue contributed at pocket classifications on the ordinate, which are based on R_inaccess_ (see text). Isologous homopairs refer to protomer pairs which contribute the same residues to the interface, i.e. the same protein sequence bound with 180° rotational symmetry.

Recently we have analysed a non-redundant set of 15,500 pairwise, non-overlapping PPI interfaces curated from the Protein Databank (PDB), from binary and higher-order complexes. We have distinguished between interactions of proteins including enzymes with peptides on the one hand, and homologous and heterologous globular interaction interfaces on the other. We compared segmentation (binding epitope continuity), solvent accessibility, secondary structure, interatomic interactions and binding depth (Jubb et al., in preparation), systematically treating each protein in turn as receptor and measuring the depth occupied by each residue using the program Ghecom (Kawabata, 2010). Ghecom measures the smallest probe size that cannot enter a cavity (R_inaccess_), as a per-residue measure of depth of occupation or formation of a binding site. Our preliminary data indicate that while protein-peptide interactions make better overall use of interface surface pockets on their protein partners compared to other classes of interaction (Fig. 3a), interactions between two globular proteins often make use of deep interaction sites (Fig. 3b), even if only via a small pocket fitting a single residue. A remaining challenge is to identify how best to utilise the depth used by PPI partner proteins in the development of chemical modulators. Pocket detection software is important for this purpose, however detection algorithms parameterised for the detection of “traditional”, large volume single pockets may miss potential, albeit more challenging sites for modulation, which are hidden in the landscape of larger protein–protein interfaces.

## 4 How flexible loops and extensions might help

If flexible peptides exploit well-defined pockets, is this also true of flexible regions in globular regions when they mediate protein–protein interactions?

**Fig. 4:**
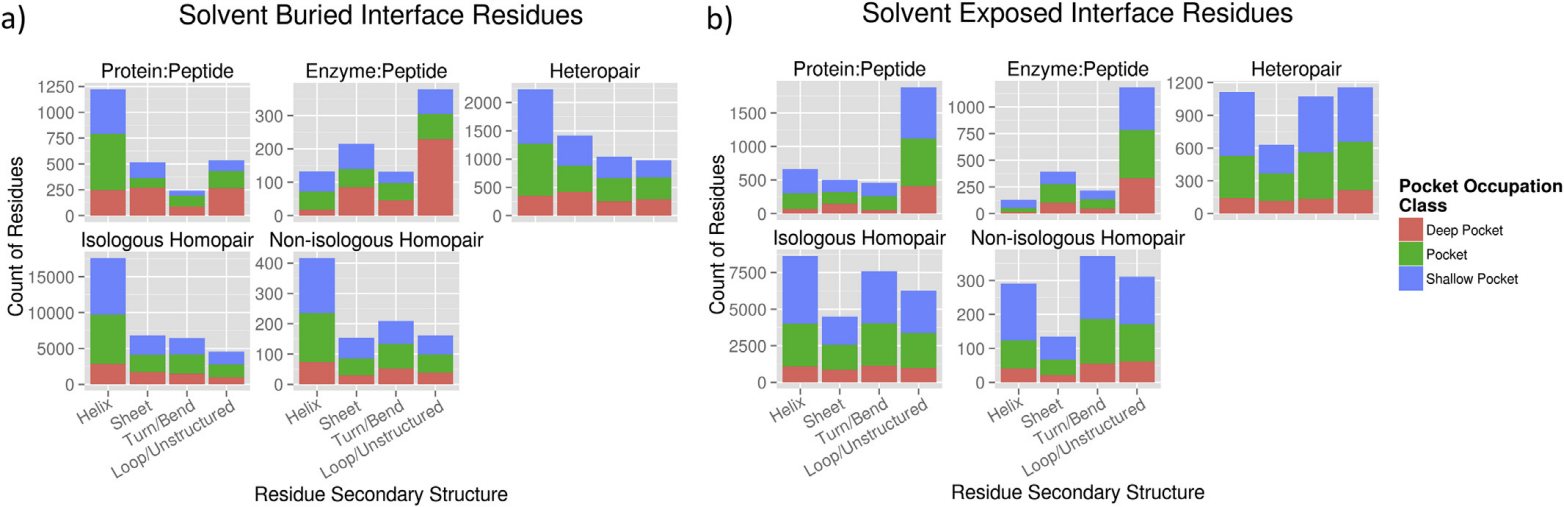
**Pocket occupation by interface residues of different secondary structures**. The secondary structure of buried (a) and solvent exposed (b) interface residues occupying concavities varies by the type of interface. Together, loop and turn regions dominate the examples of pocket bound residues in solvent exposed environments, whereas buried residues bound in pockets and grooves tend to be found in helices. Interface residue data are derived from a non-redundant subset of pairwise, non-overlapping PDB interfaces.

Preliminary analysis of the secondary structures utilised by deeply bound residues indicate that, while solvent inaccessible residues bound deep in pockets are very often found in helices, there are many examples of loop, bend and turn residues that are deeply bound (Fig. 4).

One example of the involvement of loop residues at interfaces is in camelid and nurse shark heavy chain-only (VHH) antibodies that are approximately 10 times smaller than conventional immunoglobulin G's, and lack light chains (Hamers-Casterman et al., 1993, Holliger and Hudson, 2005). The elucidation of their crystal structures has revealed framework regions and complementarity-determining regions similar to conventional immunoglobulins (De Genst et al., 2006, Desmyter et al., 1996, Spinelli et al., 1996). Interestingly, many VHH chains have longer complementarity-determining region 3 (CD3) loops (Muyldermans et al., 1994), which facilitate binding into deeper cavities not recognised by conventional antibodies (De Genst et al., 2006, Lauwereys et al., 1998, Stijlemans et al., 2004). For example, VHH antibodies have even been developed as competitive enzyme inhibitors, with the crystal structure of a VHH inhibitor of lysozyme revealing the loop inserted deep into the active pocket (Desmyter et al., 1996). The resulting interaction is a prime example of the loops within a globular protein utilising deeper pockets and anchoring the partners. These features may also occur in conventional antibodies in which the CD3 loop is longer.

It is clear that similar features may also occur in other systems where flexible loops mediate protein–protein interactions. An obvious example is in the self-association of RAD51 through loops containing the conserved FxxA repeats in nuclear protein filaments, for example defined by Shin et al. (Shin et al., 2003) in archaeal Rad51 structures and mimicked in the BRC repeats of BRCA2 (see above). We are currently analysing our structural protein–protein interaction databases to see how widespread this feature might be (Fig. 4) and whether it can provide useful clues about potentially druggable sites.

Interactions involving the termini of a protein have also been exploited in the development of peptide PPI inhibitors, for example the angiotensin II receptor antagonists mimicking Angiotensinogen (Brunner et al., 1973). Further examples of the involvement of flexible extensions in PPI are found in the binding of HGF/SF to the Met tyrosine kinase receptor (Met), which initiates a number of downstream signalling events including cell proliferation, motility, angiogenesis, morphogenesis and invasiveness (Birchmeier et al., 2003). This interaction occurs both through high- and low-affinity binding sites of the N-terminal and C-terminal regions of HGF/SF respectively to the β-propeller sema-domain of the Met receptor (Hartmann et al., 1998, Holmes et al., 2007, Kirchhofer et al., 2004, Lokker et al., 1992). The N-terminal NK1 region of HGF/SF occurs as a natural splice form and has been shown able to form a high-affinity association with Met in the presence of heparin, with the crystal structure revealing a patch of amino acid residues (Glu159, Ser161, Glu195 and Arg197) crucial for the interaction and activation of Met on either side of the homodimer (Chirgadze et al., 1999, Ultsch et al., 1998, Youles et al., 2008). These residues form a similar cavity to the lysine-binding pockets of other kringle domains and this has been proposed to mediate the dimerisation and activation of the Met receptor. Indeed current work in our laboratory shows that a highly charged segment of a loop of MET harbouring the furin cleavage site of MET (E302-E312: EKRKKR | STKKE) may contribute a secondary interface with the lysine-binding pocket of kringle 125 (Blaszczyk et al., in preparation). Intriguingly, NK1 can even be converted into a receptor antagonist of Met by mutations that alter this interface (Tolbert et al., 2007).

Interactions with the Met sema domain by the C-terminal serine-protease-like domain of HGF/SF are also likely to be the result of a combination of order–order and disorder-order binding. For example, this lower-affinity binding site of HGF/SF contains a number of residues linked to reduced Met signalling. This interaction is similar to the substrate processing region of serine proteases, with a core triad of homologous catalytic residues, a ‘hot-spot’, and interactions with the corresponding c220 activation domain loop (Stamos et al., 2004). From the crystal structure of the complex, it was observed that these regions interact with three separate loops of the Met sema domain (Stamos et al., 2004).

## 5 Chemical modulators targeting small pockets in PPIs

Several chemical methodologies designed to modulate PPI interfaces, in particular interfaces with deep pockets and grooves, including alpha-helical mimetics (Fletcher and Hamilton, 2005) and stapled peptides, target α-helical peptide molecular recognition sites (Chang et al., 2013, Raj et al., 2013). Successful target interfaces include Bcl-2/XL:BAD (Oltersdorf et al., 2005), BH3:Mcl-1 (Stewart et al., 2010), p53:MDM2/X (Bernal et al., 2010), and MAML-1:Notch (Moellering et al., 2009), all of which involve helices central to the protein–protein interactions and are likely concerted folding-and-binding interactions. Possibilities for β strands to be used as mimetic templates are also being explored (Watkins and Arora, 2014).

An analysis of different PPI revealed that for many targets it would be necessary to expand the available chemical diversity space in order to identify small-molecule PPI inhibitors (Pagliaro et al., 2004). One of the most promising strategies for the identification of small-molecule PPI inhibitors has been fragment-based drug discovery, which is an effective tool to rapidly explore a much larger chemical space. Fragment-based drug discovery involves exploration of chemical space using molecules with molecular weights <300, resulting in initial hits that bind with low affinity. As a consequence they usually do not disrupt protein–protein interfaces, unless they are tethered (Wells and McClendon, 2007). An alternative fragment-based approach is to stabilise the uncomplexed components of the multiprotein system in solution and employ biophysical methods – nuclear magnetic resonance (NMR), X-ray crystallography, surface plasmon resonance (SPR), differential scanning fluorimetry (DSF) or isothermal calorimetry (ITC) to detect fragment binding (Blundell et al., 2002, Hajduk and Greer, 2007, Murray and Blundell, 2010, Shuker et al., 1996). Stabilisation for crystallography by antibodies of otherwise rarely sampled monomer conformations has been demonstrated as an exciting tool to explore protein conformational space for drug discovery, particularly with respect to making allosteric effecting sites available to small-molecule fragment binding in crystals (Lawson, 2012). Fragment hits derived from these approaches can subsequently be evolved into larger lead-like and drug-like molecules with higher affinity and potency.

One example of a successful fragment-driven campaign against a PPI interface is the RAD51:BRCA2 interaction (introduced above). Hyvönen and coworkers have engineered a monomeric form of RAD51 by humanising a thermostable archaeal orthologue, RadA, for use in fragment screening (Scott et al., 2013). The initial fragment hits were carefully validated biophysically by ITC and NMR techniques and observed by X-ray crystallography to bind in a shallow surface pocket that is occupied in the native complex by the side chain of a phenylalanine from the conserved FxxA interaction motif found in BRCA2 (Scott et al., 2013). This represents the first report of fragments or any small molecule binding at this protein– protein interaction site, and shows that small molecules targeting hotspots can effectively target interactions involving concerted folding and binding.

In the longer term small-molecule inhibitors of PPI may become second generation, less costly alternatives to the use of antibodies to directly compete for the binding sites. One application might be to use small-molecule inhibitors to target the binding sites described above of the Met interactions with HGF/SF, so providing less expensive agents than the antibodies designed to target the HGF/SF (Cao et al., 2001) and the Met sema domain (Petrelli et al., 2006).

As experimental data on first-generation PPI inhibitors are becoming increasingly available, several databases now record information on small-molecule inhibitors of protein–protein interactions. TIMBAL (Higueruelo et al., 2013b, Higueruelo et al., 2009) integrates chemical assay information from ChEMBL (Bento et al., 2014, Gaulton et al., 2012) whereas 2P2Idb (Basse et al., 2013, Bourgeas et al., 2010) records protein–protein interfaces where the structures of both protein–protein complex and protein-inhibitor complex have been defined. Analysis of these databases opens avenues to improvement of PPI inhibitor design. For example, analysis of the TIMBAL database revealed that current orthosteric PPI inhibitors tend to be relatively large and have low lipophilic efficiency, indicating potential unsuitability for use as oral drugs (Higueruelo et al., 2012). In the pursuit of ADMET favourable inhibitors, at one of the extremities of the small molecules chemists have engineered clusters of hydrophilic regions (Kuenemann et al., 2014). Machine-learning approaches have also helped identify chemical rules to help guide design of chemical libraries for PPI screening, with the molecular shape being an important determinant and the ‘Rule of 4’ providing a rapid method to enrich a library for PPI inhibitors (Hamon et al., 2014, Neugebauer et al., 2007, Reynes et al., 2010). We hope that the new insights described in this review coupled with analysis of current and future PPI interfaces and their chemical modulators will result in more effective PPI modulators with improved molecular properties, opening the doors to more specific, targeted, safe and effective therapeutics.
